# Role of proteinase-activated receptor-2 in allergic sensitization to house dust mite allergens

**DOI:** 10.1186/1710-1492-7-S2-A30

**Published:** 2011-11-14

**Authors:** Courtney Davidson, Danny Polley, Muhammad Asaduzzaman, Narcy Arizmendi, John R Gordon, Morley D Hollenberg, Harissios Vliagoftis

**Affiliations:** 1 Pulmonary Research Group, Department of Medicine, University of Alberta, Edmonton, AB Canada; 2 Department of Pharmacology and Therapeutics, University of Calgary, Calgary, AB Canada; 3 Immunology Research Group, University of Saskatchewan, Saskatoon, Saskatchewan, Canada

## Background

A number of common aeroallergens have serine proteinase activity, which is important for allergic sensitization. House dust mite (HDM), and other allergens with serine proteinase activity activate Protease-Activated Receptor-2 (PAR-2). We have shown that PAR-2 activation in the airways leads to allergic sensitization to concomitantly inhaled antigens, implicating PAR-2 in the pathogenesis of asthma. We hypothesized that PAR-2 activation in the airways by HDM allergens is important for the development of allergic sensitization.

## Methods

HDM extract was administered to mice intranasally for 5 consecutive days to induce allergic sensitization. One group of mice received a blocking anti-PAR-2 antibody intranasally before each HDM administration.

## Results

Administration of the PAR-2 blocking antibody decreased IL-4, IL13 and IL-33 mRNA as well as IL-4, IL-5 and MIP1A protein levels in the lung tissue, suggesting decreased allergic airway sensitization. Mice sensitized in the presence of the PAR-2 blocking antibody or isotype control were then challenged intranasally with HDM extract for 4 consecutive days. Mucosal exposure to HDM extract induced AHR and airway eosinophilic inflammation. Administration of the anti-PAR-2 blocking antibody during the sensitization phase completely inhibited the development of AHR and airway inflammation in response to HDM challenge.

## Conclusions

These results indicate that HDM extract induces PAR-2-dependent allergic sensitization in mice and lead to PAR-2-dependet allergic airway inflammation. These results will allow us to better define the mechanisms of allergic sensitization to allergens with serine proteinase activity.

